# Complete Sequence of a *bla*_OXA-48_-Harboring IncL Plasmid from an *Enterobacter cloacae* Clinical Isolate

**DOI:** 10.1128/genomeA.01076-15

**Published:** 2015-09-17

**Authors:** Vera Manageiro, Margarida Pinto, Manuela Caniça

**Affiliations:** a National Reference Laboratory of Antibiotic Resistances and Healthcare Associated Infections (NRL-AMR-HAI), Department of Infectious Diseases, National Institute of Health Dr. Ricardo Jorge, Lisbon, Portugal; b CECA, ICETA, Centro de Estudos de Ciência Animal, Universidade do Porto, Porto, Portugal; c Laboratory of Microbiology, Centro Hospitalar de Lisboa, EPE, Lisbon, Portugal

## Abstract

We report a 63,584-bp conjugative IncL plasmid (pUR17313-1) from an *Enterobacter cloacae* clinical isolate, containing a *bla*_OXA-48_ gene. The plasmid sequence also carried important mobile genetic elements involved in the spread of antibiotic resistance, namely, the Tn*1999*.*2* composite transposon, which enclosed *bla*_OXA-48_-, integrase-, and transposase-encoding genes.

## GENOME ANNOUNCEMENT

Bacterial plasmids are key vectors of horizontal gene transfer, mediating the mobilization of genetic material among bacteria (1). This study aimed to characterize an IncL/M-like plasmid containing a *bla*_OXA-48_ gene from an *Enterobacter cloacae* clinical isolate, which constituted the first case of OXA-48-producing *Enterobacteriaceae* in Portugal (2).

Plasmid DNA was extracted from the transconjugant Tc17313-1 (2), using a NucleoBond Xtra Plus kit (Macherey-Nagel) according to the manufacturer’s instructions. Plasmid-Safe ATP-Dependent DNase (Epicentre) was used to eliminate any contamination with chromosomal DNA. The molecular size of the OXA-48-carrying plasmid was estimated by using a GeneRuler High Range DNA Ladder (Thermo Scientific). Five hundred nanograms of the plasmid DNA were fragmented by nebulization, followed by adaptor ligation to create double-stranded DNA libraries and pyrosequenced in GS FLX (454 Roche-Life Sciences), with Titanium chemistry, according to the manufacturer’s standard protocols. The sequencing with the 454 pyrosequencer produced 174,217 reads with an average length of 464 bases. Sequencing reads were assembled with the GS Assembler version 2.8 (Roche) into 19 contigs, the largest being 40,523 bp long.

Analysis of the coverage indicated the presence of 5 contigs with more than 1,000-fold coverage, while the remaining 14 corresponded to small-length consensus sequences with residual coverage (2.7- to 8.8-fold). The ResFinder version 2.1 tool (3) was used to detect the *bla*_OXA-48_ gene in the pUR17313-1 plasmid; no other acquired antimicrobial resistance gene was found.

The submission of the 5 contigs to BLASTn (http://blast.ncbi.nlm.nih.gov) enabled the identification of the closest plasmid sequences. Final annotation of the plasmid was performed with the NCBI Prokaryotic Genome Annotation Pipeline (http://www.ncbi.nlm.nih.gov/genome/annotation_prok). The BLASTn search identified that contigs were highly similar to the *Klebsiella pneumoniae bla*_OXA-48_-encoding pOXA-48 (JN626286), E71T (KC335143), pKPoxa-48N1 (NC_021488), pKPoxa-48N2 (NC_021502), and pKpn-E1.Nr7 (KM406491) plasmid sequences, detected in Turkey (4), France (5), Ireland (6), and Switzerland (7). Therefore, the pUR17313-1 plasmid structure was constructed based on the genetic organization of those plasmids, and the contig neighbors predicted from contig assembly information.

Overall, plasmid pUR17313-1 was 63,584 bp in length with a G+C content of 51.2%. The presence of a Tra region revealed that the plasmid was conjugative. The *bla*_OXA-48_ gene was enclosed on a Tn*1999*.*2* composite transposon. Although this plasmid was not typeable by PCR-based replicon typing (2, 4), the *inc*RNA sequence revealed that pUR17313-1 was an IncL (7). In addition, PHAST analysis predicted one putative incomplete prophage region, from position 3,856 to 17,385 (13,530 bp), consisting of 28 putative coding sequences, including procapsid-like particles and integrase- and transposase-encoding genes, with a 4.32% G+C content (8).

We confirmed that the *bla*_OXA-48_ gene was carried by the widespread 63-kb conjugative IncL plasmid, which did not encode additional resistance markers but contained other important mobile genetic elements involved in the spread of antibiotic resistance. Given the clinical and epidemiological relevance of these plasmids, its complete sequence is important to understand plasmid evolution and differentiation. In the end, the availability of complete plasmid sequences from different countries supports the global epidemiological surveillance of antibiotic resistance spread.

### Nucleotide sequence accession number.

The genome sequence of pUR17313-1 has been submitted to GenBank under the accession number KP061858.
